# Maternal delay for institutional delivery and associated factors among postnatal mothers at Southeastern Ethiopia: a cross sectional study

**DOI:** 10.1186/s12884-024-06416-z

**Published:** 2024-03-18

**Authors:** Derese Eshetu Debela, Zeleke Aschalew, Agegnehu Bante, Manaye Yihune, Degefa Gomora, Feisal Hussein, Ayele Sahile, Abera Mersha

**Affiliations:** 1 Department of Midwifery, College of Medicine & Health Sciences, Madda Walabu University, Goba, Ethiopia; 2 School of Nursing, College of Medicine & Health Sciences, Arba Minch University, Arba Minch, Ethiopia; 3 School of Public Health, College of Medicine & Health Sciences, Arba Minch University, Arba Minch, Ethiopia; 4 Department of Midwifery, College of Medicine & Health Sciences, Madda Walabu University, Shashamane, Ethiopia; 5 Department of Midwifery, College of Medicine & Health Sciences, Wolkite University, Wolkite, Ethiopia

**Keywords:** Maternal delay, Institutional delivery, Postnatal, Bale, Ethiopia

## Abstract

**Background:**

Maternal delay in timely seeking health care, inadequate health care and the inability to access health facilities are the main causes of maternal mortality in low and middle income countries. The three-delay approach was used to pinpoint responsible factors for maternal death. There was little data on the delay in decision making to seek institutional delivery service in the study area. Therefore, the aim of this study was to assess the extent of the first maternal delay for institutional delivery and its associated factors among postpartum mothers in the Bale and east Bale zones.

**Methods:**

An institutional-based cross-sectional study was conducted among 407 postpartum mothers from April 6 to May 6, 2022. A systematic sampling technique was used to select study participants. The data were collected electronically using an Open Data Kit and exported to SPSS window version 25 for cleaning and analysis. Both bivariate and multivariable analysis was done by using binary logistic regression model to identify factors associated with maternal delay for institutional delivery services. Statistical significance was declared at *P*-value < 0.05.

**Results:**

In this study, the magnitude of the first maternal delay in making the decision to seek institutional delivery service was 29.2% (95% CI: 24.9, 33.9). Previous pregnancy problems (AOR = 1.8; 95% CI: 1.06, 3.08), knowing the danger signs of labor and childbirth (AOR = 1.78; 95% CI: 1.11, 2.85) and decision-making (AOR = 0.42; 95% CI: 0.20, 0.85) were significantly associated with the first maternal delay.

**Conclusion:**

This study identified a significant number of postnatal mothers experienced delay in making decisions to seek institutional delivery service in the study area. Promoting women’s empowerment and building on key danger signs should be emphasized.

## Background

Maternal delay for institutional delivery service have a big impact on maternal mortality [1]. Globally, more than eight hundred women die every day from complications related to pregnancy and delivery. Over 295,000 women die every year, with low-income countries accounting for 95% of maternal deaths [2]. Of these, greater than two-thirds (68%) of all maternal mortality takes place in Sub-Saharan Africa (SSA) [3]. According to the Ethiopian Demographic Health Survey (EDHS) report, the maternal mortality rate (MMR) in Ethiopia is 412 deaths per 100,000 live births [4]. Nonetheless, by 2030, the global goal is to lower the ratio of maternal deaths to less than 70 per 100,000 live births, striving to realize the aim of eradicating all avoidable maternal deaths [5].

Maternal delay is identified as contributing factors to maternal morbidity and death in Ethiopia. In addition, it is also responsible for maternal morbidities such as vaginal or uterine prolapse and puerperal psychosis, which can have negative physical, mental, social and financial impacts for respective parents [6]. According to findings from 700 maternal death review reports, maternal delay one was accountable for 42% maternal deaths. Thus, the main reason for the delay one is a lack of timely decision-making to go to a health facility [7].

The magnitude of the first maternal delay for institutional delivery from the time of making the decision to seek care to starting the journey to a health facility has varied in low and middle-income countries. A study revealed that the magnitude of the first delay experienced by the mother was 12%, 39.5% and 70% respectively [8–10]. Studies conducted in Ethiopia also showed that the magnitude of mothers who experienced the first delay in the utilization of institutional delivery services ranged from 27.2 to 59.7% [6, 11–14].

The Ethiopian federal ministry of health has taken several actions to tackle the problem of unreasonable high maternal delay through various activities to enhance community demand for increased access and service utilization. By linking with health extension program initiatives such as early antenatal care initiation, pregnant women conferences, maternal waiting home service, user-fee exemption for institutional delivery service, obstetric referral network, by availing ambulance service to reach health facility for childbirth and community mobilization campaigns since July 2005 have been carried out in the country [6, 7, 15].

In spite of these initiatives to increase the number of health facility-based delivery services in the country, only 48% of births were attended by trained birth attendants [16]. Trained birth attendants play a crucial role in decreasing maternal and neonatal deaths because they give timely obstetric and newborn care for life-threatening complications [15]. According to available evidence, poor knowledge of danger signs at labor and childbirth, home deliveries by untrained birth attendants, inadequate birth preparedness and its complication readiness, the decision-making powers of women, illiteracy, high cost and long distance were factors that contributed to maternal delay one [7, 15, 17, 18].

Earlier studies conducted in the country found factors linked to maternal delay in obtaining institutional delivery services. However, most of these studies were done before the coronavirus disease 2019 (COVID-19) outbreak, which posed a significant challenge to the health care delivery system. Besides, there was little information about maternal delay in utilizing institutional delivery services in the study area. Furthermore, it was crucial to explore new variables of maternal delay in seeking delivery services. Therefore, the intention of this study was to ascertain the extent of maternal delay for institutional delivery and associated factors among postnatal mothers at public hospitals of Bale and East Bale zones, Oromia region, Ethiopia.

## Methods

### Study design, period and settings

A facility-based cross-sectional study was conducted in the six public hospitals located in the Bale and East Bale zones, Oromia region, Ethiopia, from April 6th to May 6th, 2022. The Bale and East Bale zones were found in the Oromia regional state in southeastern Ethiopia. The zone was made up of twenty-one districts, of which nine were agrarian, nine were agro-pastoralist and three were town administrations. There were 1,888,366 people living in the zones, of which 936,630 were female and 65,526 deliveries were expected for the year.

The zones contained a total of six public hospitals, namely: Delo Mena General Hospital, Ginir General Hospital, Goro Primary Hospital, Madda Walabu Primary Hospital, Madda Walabu University Goba Referral Hospital and Robe General Hospital. All hospitals provide complete emergency, obstetric and neonatal care services [19].

### Population

All women who gave birth in the public hospitals of Bale and East Bale zones were regarded as source of population while all women who gave birth during the data collection period in the public hospitals of Bale and East Bale zones were considered the study population and the study unit was made up of women who gave birth in the hospitals.

### Eligibility criteria

All women who gave birth in the Bale and East Bale zones of public hospitals were included in the study, while women who unable to speak or hear and utilized a maternal waiting home during the data collection period were excluded.

### Sample size and sampling procedure

The sample size required for this study was calculated using the Epi-Info version 7.2.5 statistical software package with the assumptions of a 95% confidence level (Zα/2 = 1.96), 5% margin of error (d = 0.05) and the proportion of mothers who experienced delays during emergency obstetric care was 59.7% [14]. Considering a 10% non-response rate, the final sample size required for this study was found to be 407postnatal mothers.

All hospitals found in the Bale and East Bale zones were included. The average source population of each hospital was determined by reviewing the previous year’s similar-month delivery report during a data collection period. The proportional allocation was done for six hospitals to collect 407 samples. The study participants were systematically selected with an interval of K every three persons from each hospital. Eventually, the total sample size needed was collected within the specified period of time.

### Data collection tools and procedures

The structured questionnaire that was carried out by an interviewer was adapted from the survey tools developed by the Johns Hopkins Program for International Education in Gynecology and Obstetrics (JHPIEGO) maternal and neonatal health program [20] and from prior literature reviews[6, 11–14, 21]. The questionnaire contains three parts: socio-demographic and economic factors, Obstetric related factors and delay in making decisions for institutional delivery related variables. A pre-test was conducted in Dodola general hospital (found in the west Arsi zone) on 21 respondents (5% of the sample size) that had similar characteristics to the study population to check wording, clarity, skip patterns and culturally sensitive concerns of the questions.

The questionnaire was collected by ODK (Open Data Kit) collect. Three bachelor degree health officers were employed as supervisors and six nurses with bachelor’s degrees were recruited for data collection. The purpose of the study was outlined and training was given for supervisors and data collectors for two days on how to use the ODK application, connect with the server, save and send files.

On the data collector’s smartphone, the ODK collect version 1.17.2 software was installed and the blank form was downloaded from the server. The tool was pre-tested a week prior to the actual data collection period. The supervisors frequently checked on the data collection process by staying in contact with the data collectors. The data was collected through face-to-face interviews with postnatal mothers in private rooms while they were discharged.

### Study variables

#### Dependent variable

First maternal delay.

#### Independent variables

Socio-demographic related factors such as age, residence, marital status, education, occupation head of household and income. Obstetrics related factors: gravidity, parity, antenatal care follow-up, frequency of antenatal care, previous pregnancy-related problems, women’s knowledge on danger signs of labor and childbirth, birth readiness, type of delivery, mode of delivery, chronic illness, current pregnancy planned and wanted, place of antenatal care, problem in current pregnancy, place of delivery, consultation about emergency obstetric care, labor start and decision making.

### Operational definitions and measurement

The first maternal delay was the time interval between the recognition of the labor and make decision to seek institutional delivery service. Time taken ≥ 1 h to make a decision to seek care was considered as delay and less than an hour was considered no delay [6]. Emergency obstetric care: emergency medical or surgical attention given to a woman who was in labor or had recently given birth [11]. Institutional delivery service: when a mother gave birth in a health facility and was assisted by a skilled birth attendant [6].

Non-referred mothers: a mother who gave birth among selected hospitals and had not been referred from another health care facility to the selected hospital for advanced care [14]. Knowledgeable of key danger signs during labor and childbirth: If a woman spontaneously mentioned four major danger signs for labor or childbirth which were convulsions, severe vaginal bleeding, retained placenta and prolonged labor ( >12 hours) [22].

### Data quality control

The structured questionnaire was initially developed in English and translated into Amharic and Afan Oromo versions by language experts, then back to English for consistency. The training was provided to data collectors and supervisors on how to collect data using the ODK Collect application. A pretest was done to ensure the clarity of the tool. The supervisors checked and reviewed the questionnaires to ensure the completeness of the forms. Each woman was interviewed in a separate private place to avoid social desirability bias. Moreover, the investigators were kept in touch with the server to regularly check the sent files from each data collector.

### Data processing and analysis

The data collector’s smart mobile phone sent each data file to the server. The file was downloaded from the server and saved as an Excel file. The data set was then imported to SPSS 25.0 versions for cleaning, coding and analysis. Descriptive statistics such as frequency, percentage, mean and standard deviation were computed to describe the characteristics of participants. Bivariate and multivariable analysis were done in the binary logistic regression model to identify factors. The assumptions of the binary logistic regression model were checked.

A *P*-value < 0.25 in the bi-variate analysis was considered to took a candidate variable for the final model. The hosmer-lemeshow goodness fitness was done to check model fitness. Multicollinearity among independent variables was checked by variance inflation factor (VIF < 10). The adjusted odds ratio (AOR) with a 95% confidence interval (CI) was computed to determine the level of significance. A statistical significance was declared at *P*-value < 0.05. The result was presented by using tables and figures.

## Results

### Socio-demographic and economic characteristics

The present study included 407 postnatal mothers who were voluntarily involved. Of the study participants, three hundred ninety seven (97.5%) were married and two hundred eighty-one (69%) were housewives. One hundred forty-four (35.4%) had no formal education. In this study, 190 (46.7%) mothers were rural residents. The mean (±SD) age of the participants was 27.34 (±6) years. Of the study respondents, two hundred fifty-five (62.6%) were within the age group of 21–34 years (Table 1).

**Table 1:** Socio-demographic and economic characteristics of participants in the public hospitals of Bale and East Bale zones, Oromia, Ethiopia, 2022 (*N* = 407)

Variables	Frequency	Percentage (%)
**Residence**		
Urban	217	53.3
Rural	190	46.7
**Age(in completed years)**		
15–20	74	18.2
21–34	255	62.6
≥ 35	78	19.2
**Women Education**		
Illiterate	144	35.4
Primary(Grade 1–8)	154	37.8
Secondary (Grade 9–12)	64	15.7
Tertiary and above	45	11.1
**Marital status**		
Married	397	97.5
Others^β^	10	2.5
**Women Occupation**		
Government Employee	41	10.1
House wife	281	69
Private	73	17.9
Other^α^	12	3
**Husband’s Education**		
Illiterate	101	24.8
Primary (grade 1–8)	152	37.3
Secondary(grade 9–12)	80	19.7
Tertiary and Above	74	18.2
**House hold average monthly Income (ETB)**		
Less than or equal to 1000	114	28
1001–1999	36	9
Greater than or equal to 2000	257	63
**Head of house hold**		
Woman herself	41	10
Joint	73	18
Husband	293	72
**Husband’s Occupation**		
Private	166	40.8
Farmer	169	41.5
Government employee	72	17.7

^α^ -Farmer, Students, ETB-Ethiopia Birr ^β−^single, divorced, widowed

### Obstetrics care related characteristics

 Two hundred ninety three (72%) postnatal mothers were multipara and ninety eight (33.4%) mothers had obstetric problems in previous pregnancies. Seventy three (17.9%) postpartum mothers had a past chronic disease during the current pregnancy. The current pregnancy was planned for 293 (72%) and it was wanted for 300 (73.3%) of the study participants.

Among the respondents, 340 (83.5%) had initiated antenatal care follow-up and 137 (33.7%) had faced obstetric problems in the current pregnancy. The health center was the place of antenatal care follow-up for 169 (49.7%) mothers and 183 (53.8%) had fewer than four visits.

Two hundred twenty (54.1%) postnatal mothers had poor knowledge of danger signs during labor and delivery. Moreover, the final decision to seek institutional delivery service was made by husbands for 207 (50.9%) mothers (Table 2).

**Table 2:** Obstetric characteristics of participants in the public hospitals of Bale and East Bale zones, Oromia, Ethiopia, 2022 (*n* = 407)

Variable		Frequency	Percentage (%)
**Gravidity**			
Primigravida		114	28.0
Multigravida		293	72
**Antenatal Care follow up**			
No	67		16.5
Yes	340		83.5
**Women knowledge on danger signs of labor and childbirth**			
Poor	220		54.1
Good	187		45.9
**Readiness to deliver in health facility**			
No	89		21.9
Yes	318		78.1
**Labor start**			
Night	247		60.7
Day	160		39.3
**Final decision maker**			
Woman herself	121		29.7
Husband	207		50.9
Joint	79		19.4
**Consultation about Emergency Obstetric Care**			
Families	84		20.6
Traditional Birth Attendants	18		4.4
Health workers	305		75
**Current mode of delivery**			
Non Spontaneous Vaginal delivery	166		40.8
Spontaneous Vaginal delivery	241		59.2

### Magnitude of maternal delay and reasons

In this study, the magnitude of the first maternal delay, delay in making decision to seek institutional delivery service was 29.2% in the study area. This study found that 47 (11.5%) mothers had delayed seeking care after realizing that labor had begun. They complained that 38 (9.3%) feared childbirth at a health facility, 36 (8.8%) COVID 19, 26(6.4%) failed to recognize labor symptoms, 20 (4.9%) lack of transportation, and 9 (2.2%) distance.

### Factors associated with first maternal delay among postnatal mothers

In bi variable analysis, residence, mother’s education, husband’s education, previous pregnancy problem, planned pregnancy, ANC follow up, number of ANC, problem in current pregnancy, mother’s knowledge of labor and childbirth, consultations for institutional delivery and final decision maker were statistically significant at a *P* value less than 0.25. In multivariable analysis, previous pregnancy problems, knowledge of danger signs during labor and childbirth and decision-making for institutional delivery service were found to be statistically significant at *P* < 0.05 with the first maternal delay.

The women who faced no obstetric problems during the previous pregnancy were 1.8 times more likely to have the first delay in the decision to seek institutional delivery service (AOR = 1.8 (95%CI:1.06,3.08) than those who faced obstetric problems. The odds of the first delay in seeking institutional delivery service among women who didn’t know the danger signs during labor and childbirth were 1.78 (AOR = 1.78, 95%CI: 1.11, 2.85) as compared to counterparts. The mothers who had made the decision jointly with their husbands were 58% less likely (AOR = 0.42, 95% CI: 0.20, 0.85) delayed in seeking institutional delivery service than those who had decided alone (Table 3).

**Table 3:** Factors associated with first delay among postnatal mothers in the public hospitals of Bale and East bale zones, Oromia, Ethiopia,2022(*n* = 407)

Variables	First maternal delay		COR[95%CI]	AOR [95%CI]	*P* value
	Yes	No			
**Residence**					
Rural	48(25.3%)	142(74.7)	0.69(0.45,1.07)	0.69(0.44,1.09)	0.118
Urban	71(32.7%)	146(67.3)	1	1	
**Previous pregnancy problem**					
Problem	83 (26.9%)	226(73.1)	1	1	
No problem	36 (36.7%)	62(63.3%)	1.58(0.98,2.56)	1.8(1.06,3.08)*	0.031
**Planned pregnancy**					
No	40(35.1%)	74(64.9%)	1.46(0.92,2.33)	1.15(0.7,1.87)	0.588
Yes	79(27%)	214(73%)	1	1	
**Problem in the current pregnancy**					
Yes	46(33.6%)	91(66.4%)	1.36(0.87,2.13)	1.24(0.77,2)	0.369
No	73(27%)	197(73%)	1	1	
**Mother’s knowledge of labor and childbirth**					
No	76 (34.5%)	144(65.5)	1.77(1.14,2.74)	1.78(1.11,2.8)*	0.017
Yes	43(23%)	144(77%)	1	1	
**Final decision Maker**					
Husband	60(29%)	147(71%)	0.71(0.44,1.15)	0.78(0.47,1.29)	0.345
Joint	15(19%)	64(81%)	0.41(0.21,0.80)	0.41(0.2,0.85)*	0.016
Woman	44(36.4%)	77(63.6%)	1	1	
**Consultation about emergency obstetric care**					
Health worker	85(27.9%)	220(72.1)	1	1	
Family	30(35.7%)	54(64.3%)	1.44(0.86,2.39)	1.16(0.67,2.02)	0.592
TBA	4(22.2%)	14(77.8%)	0.74(0.24,2.31)	0.78(0.24,2.59)	0.695

(* = a statistically significant variable at *p* < 0.05)

## Discussions

The findings of this study revealed that the magnitude of the first maternal delay among postnatal mothers at public hospitals of Bale and east Bale zones was 29.2% (95% CI: 24.9–33.9). Pregnant mothers who have had previous pregnancy problems, knowledge about danger signs during pregnancy and childbirth and decision-making for institutional delivery services were found to be significantly associated with delay one.

The findings of this study was consistent with a study carried out in the Arsi zone 26.2% [11]. This might be because the study respondents shared similar sociodemographic and economic characteristics, culture and way of life as they were living in neighbouring zones. This implies that there was a delay at the community level.

On the other hand, the current finding was higher than the study done in North Shewa 23% [23]. A possible reason could be the mothers’ lack of awareness to inform the whole family about the danger signs of pregnancy. In the population studied, 35.4% of the mothers and 24.8% of the spouses were illiterate. In addition to this, in the present study, 46.8% of the respondents were rural residents with low socioeconomic status. This can affect the usage of the delivery services and account for a high percentage. Also, it could be due to a lack of skilled healthcare providers and a disrespectful service delivery system.

However, the delay in deciding to seek institutional delivery service was lower than in a study conducted in South Gondar 36.3% [14], Dawuro 42% [12], Jimma 46.7% [13], Ilubabor 51% [24] and Burundi 39.7% [9]. The possible explanation might be the differences in sociocultural characteristics of study participants, study period and the fact that maternal health education has progressed from time to time.

The current study demonstrated that previous pregnancy problems were a statistically significant factor with outcome variable. Those women who had no obstetric problems during their previous pregnancies were twice as likely to delay in making decision to seek institutional delivery service compared to those who had obstetric problems. This finding was supported by the study conducted in the Southern Nation, Nationalities and People region (SNNP) [6]. This might be a result of women’s developing confidence and experience that the index pregnancy would also be without problems.

The findings of this study also showed that women who had poor knowledge of the danger signs of labor and childbirth were more likely to experience a delay in seeking institutional delivery service as compared to those who had good knowledge. This result is consistent with the study conducted in Ethiopia [6]. However, this is inconsistent with the study done in the SNNP region [21]. This would be due to women’s poor awareness of obstetric danger signs during labor, delivery and variations in the period of the study.

Lastly, a delay in seeking institutional delivery service was influenced by the women’s decision-making ability. Women who made decisions jointly with their husbands were 58% less likely to experience a first delay in using institutional delivery service when compared to those who made decisions on their own. This might be interaction between the women and their husbands most certainly influences their decision to travel to institutional delivery care earlier rather than later. This implies that the husband may be able to assist her by paying for her travel expenses. On the other hand, this indicated that there is a maternal delay in seeking for institutional delivery service at the family level.

The strength of this study was the use of primary data by directly interviewing study participants and data was collected using the ODK Collect application, which increased the completeness, accuracy and quality of the data. The variable was managed independently to control the effect of confounders that prevented bias from being introduced at the analysis stage.

However, it has some limitations. Since delays are measured in time based on client responses’ estimations, they can be overestimated or underestimated. Besides, since an interviewer-administered questionnaire was used to collect data, it may be subject to recall bias. Furthermore, this study employed a cross-sectional design that failed to differentiate between cause-effect relationships.

## Conclusions

This study identified a significant number of mothers experienced delay in making decisions to seek institutional delivery service in the study area. In general, in order to alleviate maternal delay, health extension workers, together with professionals from health centers, hospitals, woreda officers, and programmers, should prioritize creating awareness, promoting income-generating mechanisms and empowering women to make the decisions that need to be strengthened and expanded in the community.

## Data Availability

The datasets generated and /or analyzed during the current study are not publicly available due to preserving participant anonymity but are available from the corresponding author on reasonable request (Derese Eshetu, dereseeshetu12@gmail.com).

## Abbreviations

ANC: Antenatal Care
COVID19: Coronavirus Disease 2019
EDHS: Ethiopia Demographic Health Survey
EMDHS: Ethiopia Mini Demographic Health Survey
MMR: Maternal Mortality Ratio
ODK: Open Data Kit
SNNP: Southern Nation, Nationalities and Peoples Region
SSA: Sub-Saharan Africa
